# Barcoding chemical modifications into nucleic acids improves drug stability *in vivo*

**DOI:** 10.1039/c8tb01642a

**Published:** 2018-08-28

**Authors:** Cory D. Sago, Sujay Kalathoor, Jordan P. Fitzgerald, Gwyneth N. Lando, Naima Djeddar, Anton V. Bryksin, James E. Dahlman

**Affiliations:** a Wallace H. Coulter Department of Biomedical Engineering , Georgia Institute of Technology , Atlanta , GA 30332 , USA . Email: james.dahlman@bme.gatech.edu; b Parker H. Petit Institute for Bioengineering and Bioscience , Georgia Institute of Technology , Atlanta , GA 30332 , USA

## Abstract

The efficacy of nucleic acid therapies can be limited by unwanted degradation. Chemical modifications are known to improve nucleic acid stability, but the (i) types, (ii) positions, and (iii) numbers of modifications all matter, making chemically optimizing nucleic acids a combinatorial problem. As a result, *in vivo* studies of nucleic acid stability are time consuming and expensive. We reasoned that DNA barcodes could simultaneously study how chemical modification patterns affect nucleic acid stability, saving time and resources. We confirmed that rationally designed DNA barcodes can elucidate the role of specific chemical modifications in serum, *in vitro* and *in vivo*; we also identified a modification pattern that enhanced stability. This approach to screening chemical modifications *in vivo* can efficiently optimize nucleic acid structure, which will improve biomaterial-based nucleic acid drugs.

## Abstract 2

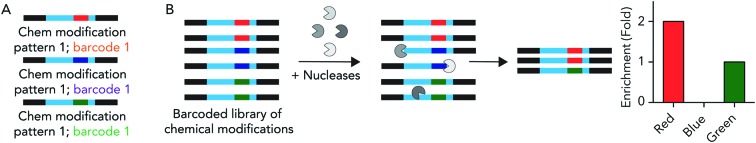
The efficacy of nucleic acid therapies can be limited by unwanted degradation.

## Introduction

Nucleic acids are therapeutic biomolecules that can be used to specifically manipulate genes in patients.^1^ There are 2 significant problems that slow the development of nucleic acid therapies. First, systemic delivery to non-hepatocyte cell types remains a significant challenge.^2^ As a result, high doses of drugs must be administered, which can cause off-target effects. Second, the nucleic acid can activate the immune system and be degraded. Nucleic acids trigger cellular sensors (*e.g.* TLRs, RIG-I), and are degraded by nucleases in serum and in cells. Nucleic acid degradation occurs through multiple mechanisms, most notably endonucleases, exonucleases, and hydrolysis. Endonucleases can cleave nucleic acids in either random or a sequence-defined manner and often are active against double-stranded nucleic acid regions. Exonucleases can degrade from single or double stranded nucleic acid termini in the 3′ or 5′ directions; it has been postulated that 3′-exonucleases are the primary cause of nucleic acid degradation in serum and plasma.^3^,^4^ However, 5′ exonuclease activity has been observed against exogenous^4^ and endogenous nucleic acids.^5^ Taken together, these 2 limitations mean potential nucleic acid drugs need to be frequently administered at high doses; this makes them impractical.

Chemical modifications are used to increase nucleic acid stability by preventing nuclease degradation and immunostimulation. Although there are many different chemical modifications, they can roughly be subdivided into modifications to the ribose (most often on the 2′ position) and modifications to the phosphodiester backbone. We set out to study how 2 commonly used chemical modifications to the 2′-ribose (2′-*O*-methyl) and phosphodiester linkage (phosphorothioates) enhance DNA stability. 2′-*O*-Methyl and phosphorothioates modifications are commonly used to enhance the biological efficacy of nucleic acids including siRNA,^6^ anti-sense oligonucleotides,^7^ DNA repair templates,^8^ CRISPR-Cas9^9^–^11^ sgRNAs, and Cpf1 crRNAs,^12^ and DNA origami.^13^ However, the (i) number, (ii) location, and (iii) type of chemical modifications affect nucleic acid stability; this makes optimizing the chemical structure a combinatorial problem. How these 3 variables interact with one another is unknown; for example, do the (i) number of ideal modifications stays consistent regardless of the (iii) type of modifications? An ideal approach would entail testing all possible chemical modification patterns 1 by 1. However, this approach would expensive and time consuming, particularly if the studies are performed *in vivo.* One unexplored alternative is to use a DNA barcode to test several chemical modification patterns simultaneously. DNA barcodes have been used to study cell lineage,^14^ resistance to cancer drugs,^15^,^16^ primary tumor growth,^17^,^18^ metastasis,^19^ viral delivery,^20^ and nanoparticle targeting.^21^–^23^ However, whether DNA barcoding can be used to optimize chemical modifications remains unexplored.

## Results

**Fig. 1:**
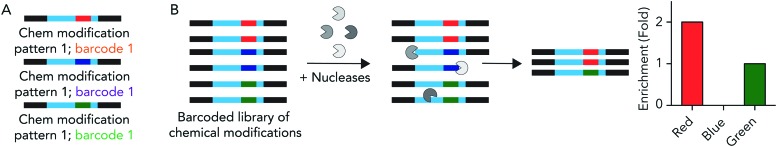
DNA barcodes can be used to study nucleic acid modifications. (A) DNA barcodes were designed such that different 8 nucleotide barcode region corresponded to different chemical modifications on the 5′ and 3′ ends of the DNA barcode. (B) We administer all the barcodes simultaneously; nucleic acids are degraded at differential rates. By sequencing the barcodes after degradation has occurred, we test how many chemical modifications improve stability at once.

We hypothesized that rationally designed DNA barcodes could be used for high throughput studies of nucleic acid modifications. To test this hypothesis, we reengineered a DNA barcoding system we previously described.^21^,^22^ In these previous studies, DNA barcodes were used to track how hundreds of chemically distinct lipid nanoparticles (LNPs) targeted cells directly *in vivo.* In the current case, we modified the DNA barcode so it tracked the chemical modification (instead of the LNP), and used the barcodes to quantify DNA degradation in serum, cells, and *in vivo.* Briefly, chemical pattern 1 was associated with barcode sequence 1; chemical pattern N was associated with barcode sequence N (Fig. 1A). We then administered the barcodes simultaneously, applying a degradation selection pressure by exposing the barcodes to serum, cells, or injecting them *in vivo.* We then performed deep sequencing to study how all the barcodes survived the degradation pressure at once (Fig. 1B).

**Fig. 2:**
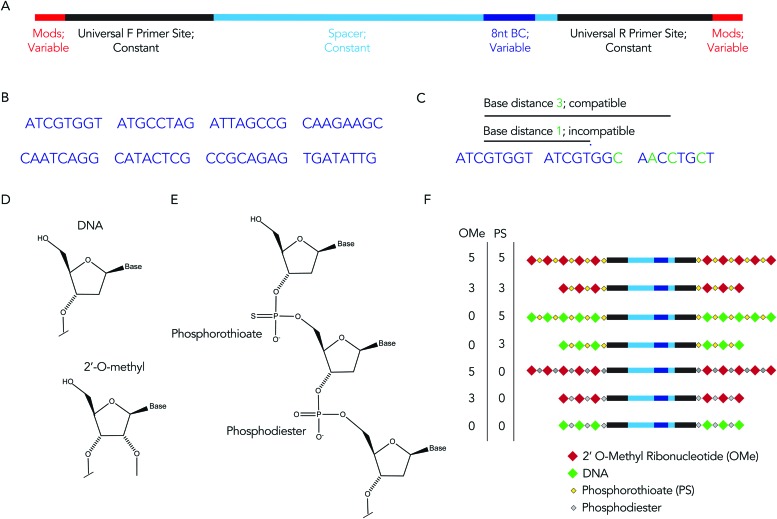
The DNA barcode is rationally designed. (A) Diagram of DNA barcode – containing universal primer sites, spacer regions, and an 8 nucleotide barcode sequence. Most of the barcode is made of constant regions, which do not change barcode to barcode (B) we used 8 different barcode sequences. Each barcode region was 8 nucleotides. (C) The 8 nucleotide sequences were designed with a base distance of 3 or more, so all barcodes could be sequenced at once. (D) DNA and 2′-*O*-methyl ribose nucleotides. (E) Diagram of DNA containing both phosphorothioate and phosphodiester linkages. (F) Diagram of 7 chemical modification patterns used in this study.

We rationally designed the barcode sequence for these studies (Fig. 2A). Briefly, each DNA sequence was identical except for 2 locations: the chemical modifications at the termini, and the DNA barcode region – which was used as the tag for that chemical modification pattern – in the middle. The ‘barcode region’ was only 8 nucleotides; this can generate 65 536 (4^8^) different barcode sequences. Of these 65 536 sequences, we selected 8 sequences with a base distance of 3 or more (Fig. 2B); in other words, every barcode was different from all other barcodes at 3 of the 8 positions (or more) (Fig. 2C). Using an Illumina QC score of 30, this ensures the odds of the sequencing calling a ‘false’ barcode is less than 1/10^9^. We have designed an additional 150 sequences with base distances of 3 or more. Flanking these variable regions – which varied with the chemical modifications – were constant regions that did not change with modification pattern. Identical forward and reverse primer sites, a well as constant spacer sequences, were included in every barcode. Chemical modifications were not incorporated in the primer sites, since chemical modifications change the melting temperature of primers, leading to biased PCR amplification. We also included small semi-randomized NWNH sites to reduce bias generated on Illumina sequencing machines. This rational design allowed us to track multiple chemical modifications using a single set of primers, ensuring all the barcodes are amplified without introducing bias.

We used these barcodes to test whether chemical modifications improve nucleic acid stability. We modified the 7 barcodes with different terminal phosphorothioates linkages and/or 2′-*O*-methyl ribose modifications, varying the number and type of modifications (Fig. 2D–F). We then exposed them to 10% mouse serum at 37 °C for either 1, 3, or 24 hours. We included a barcode without modifications as a negative control and saved the pooled DNA we added to the serum (*i.e.*, the DNA ‘input’) as an additional control. We then extracted DNA from the serum, amplified it using universal primers, and deep sequenced it using Illumina NextSeq. By comparing the amount of each barcode after exposure to serum to the amount of barcode we administered, we calculated the barcode enrichment. This ‘enrichment’ is a readout of how well chemical modification pattern 1 did, relative to chemical modification pattern 2. High enrichment meant the barcode was more resistant to degradation than the other barcodes (Fig. 1B); this readout is analogous to counts per million on RNAseq,^24^ and to normalized delivery, which we previously described.^21^,^22^

**Fig. 3:**
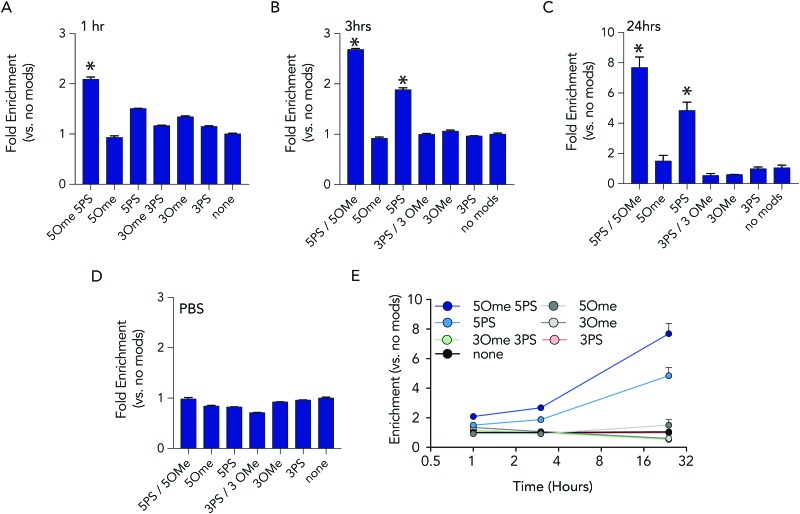
Barcodes with 5 phosphorothioates and 5 2-*O*-methyl modifications exhibit increased stability in serum. Enrichment of each DNA barcode compared to barcodes with no modifications in 10% mouse serum at (A) 1 hours, (B) 3 hours, and (C) 24 hours. (D) As one control, we measured enrichment in PBS at 24 hours, and found no differences. (E) Enrichment of the top 2 barcodes increased over time.

At each time point in the serum incubation time course the most heavily modified sequence – with 5 2-*O*-methyl and 5 phosphorothioate modifications on both the 5′ and 3′ termini – resisted degradation more than other barcodes (Fig. 3A–C). After 24 hours of incubations, the barcodes with 5 2-*O*-methyl and 5 phosphorothioate modifications were enriched by 7.7 fold, relative to the unmodified barcode; after 1 hour, enrichment was only 2 fold. DNA barcodes with just 5 phosphorothioate modifications also tended to outperform other modifications. At the 24 hour timepoint, the barcode with just 5 phosphorothioate modifications were enriched by 4.8 fold. Several control conditions that led us to believe the data were robust. First, barcodes with other modifications were not enriched relative to the control. In addition, when we added the barcodes to PBS – which should not degrade the barcodes – no enrichment was observed (Fig. 3D). Finally, we observed that the enrichment of the barcodes with either 5 2-*O*-methyl and 5 phosphorothioates and the barcodes with just 5 phosphorothioates increased with time (Fig. 3E), which we would expect if the other barcodes were being degraded.

**Fig. 4:**
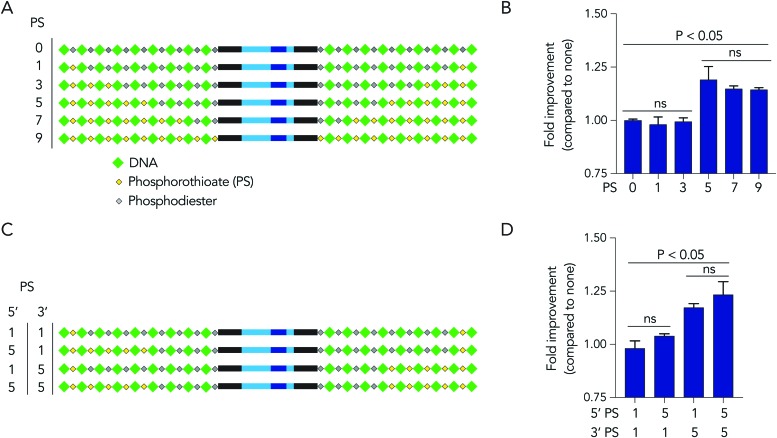
DNA barcodes can elucidate mechanisms of degradation. (A) DNA barcodes were designed with either 0, 1, 3, 5, 7, or 9 phosphorothioates, and the (B) enrichment of DNA barcodes with varying numbers of phosphorothioates in 10% mouse serum was quantified. (C) DNA barcodes were designed with unbalanced number of phosphorothioates of 5′ and 3′ termini to investigate the ‘directionality’ of degradation. (D) Enrichment of DNA barcodes with unbalanced number of phosphorothioates compared to those with balanced number of phosphorothioates.

Based on the observation that 5 modified nucleotides improved stability relative to 3 nucleotides (and none), we investigated how many phosphorothioates are needed to maximize serum stability. We designed similar DNA barcodes containing between 0 to 9 phosphorothioates on each termini (Fig. 4A), and incubated them in 10% mouse serum for 24 hours. We observed that DNA barcodes with 5, 7, and 9 phosphorothioate modifications performed similarly, and were enriched relative to barcodes with 0, 1, or 3 phosphorothioate modifications (Fig. 4B). These data suggest that 5 nucleotide modifications is minimal number that is sufficient for preventing nucleic acid degradation in human serum for 24 hours. This study design can define the minimum number of modifications required in different physiological conditions.

We then investigated whether DNA degradation occurred primarily from the 5′ or 3′ termini by designing DNA barcodes with ‘unbalanced’ modification patterns. More specifically, we designed DNA barcodes with 5 phosphorothioates on one termini and 1 phosphorothioate on the opposite termini (Fig. 4C). We hypothesized that if degradation occurs primarily on the termini with 5 phosphorothioates, the unbalanced DNA barcode would enrich; however, if degradation occurs primarily from the termini with only 1 phosphorothioate, the DNA barcode would not enrich. We observed that DNA barcodes with 5 phosphorothioates on the 3′ termini enriched compared to those with 5 phosphorothioates on the 5′ termini (Fig. 4D). These data indicate that serum DNA degradation can occur more readily from the 3′ termini.

**Fig. 5:**
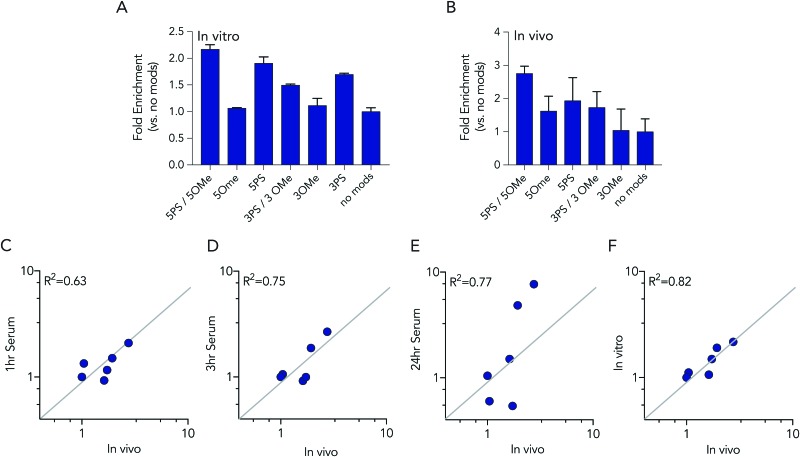
Barcodes with 5 phosphorothioates and 5 2-*O*-methyl modifications exhibit increased stability in cells and in mice. (A) Enrichment of each DNA barcode compared to barcodes with no modifications *in vitro* in HEK293T cells at 24 hours. (B) Enrichment of each DNA barcode compared to barcodes with no modifications *in vivo* from lung endothelial cells at 24 hours. Correlation of *in vivo* enrichment compared to (C) 1 hour, (D) 3 hours, (E) 24 hours incubation in 10% mouse serum, and (F) *in vitro* in HEK 293T cells.

In these initial serum experiments, we observed that barcodes containing 3 phosphorothioate or 3 2-*O*-methyl modifications were not enriched, compared to unmodified control barcodes. However, our results were in generated serum, which may not predict nucleic acids degradation in cells or in mice. Since serum assays are regularly used to study nucleic acid stability, we tested the biological relevance of the serum incubation by comparing the same pool of barcodes to cells and to mice. Specifically, we administered a pool of DNA barcodes to HEK293T cells *in vitro* using Lipofectamine 2000. Twenty-four hours later, we isolated barcodes from the cells and performed deep sequencing. Once again, we found barcodes with 5 2-*O*-methyl and 5 phosphorothioate modifications outperformed all other barcodes. The enrichment was lower; only 2.2 fold, relative to the unmodified control barcode (Fig. 5A). Barcodes containing 5 phosphorothioates were also enriched again, but to a lesser extent (1.9 fold) than they were in serum. Based on these results, we performed an additional experiment to simultaneously evaluate how all 7 barcode modifications affected stability *in vivo.* We formulated all 7 DNA barcodes into the same LNP; this LNP was previously shown to deliver small nucleic acids to pulmonary endothelial cells *in vivo*.^25^ We then administered the LNP intravenously at a dose of 0.5 mg kg^–1^, waited 24 hours, and isolated pulmonary endothelial cells using fluorescence activated cell sorting as we previously described.^21^,^25^ The results were consistent, with the 5 2-*O*-methyl and 5 phosphorothioate barcode performing the best, and the 5 phosphorothioate barcode performing the second best. They were enriched by 2.8 and 2.0 fold, respectively (Fig. 5B). In the serum, *in vitro*, and *in vivo* experiments, the unmodified control barcode was never enriched, as we expected.

We investigated whether serum or *in vitro* conditions accurately predicted nucleic acid degradation *in vivo.* Specifically, we quantified the *R*^2^ coefficient between the enrichment for in the *in vivo* experiment and serum or *in vitro* experiments. We found that degradation in cells was more likely to predict degradation *in vivo* (Fig. 5C–F). The *R*^2^ value for the serum samples was relatively high (between 0.63 and 0.75), but many of the data points were far away from the diagonal, suggesting cell assays are a better way to predict *in vivo* degradation of nucleic acid therapies. Taken together, these data demonstrated that barcodes can be used to simultaneously analyze the stability of many modified nucleotides at once, *in vitro* and *in vivo*, and also demonstrate that studying degradation *in vitro* may be a better way to predict degradation *in vivo.*

## Discussion

This work describes a novel use of DNA barcodes to analyze the impact on resistance to degradation of DNA modifications. We characterized how 2 commonly used chemical modifications (2-*O*-methyl ribose and phosphorothiate linkages) impact resistance to degradation in mouse serum at multiple time points as well as *in vitro* and *in vivo*. We found that across all conditions, DNA barcodes with 5 phosphorothiate modifications enrich compare to other modification patterns compared to DNA barcodes with fewer phosphorothioate modifications. The inclusion of 5 2-*O*-methyl modifications furthered the enrichment of barcodes already containing 5 phosphorothiate linkages, but provided little enrichment with phosphodiester linkages. Nucleotides with 5, 7, or 9 modifications tended to perform similarly, which suggested that at 24 hours in human serum, 5 modifications was the ‘minimally sufficient’ number to prevent nucleic acid degradation. It will be interesting to study this ‘minimal’ number in different physiological conditions and disease states. Notably, DNA barcodes with 0, 2, and 3 phosphorothioate modifications have been successfully applied *in vivo* for the study of the impact of chemotherapeutics^23^ and nanoparticle biodistribution.^21^ This work can inform the design of future DNA barcodes with increased stability *in vivo*. One important limitation is that we did not study additional chemical modifications (*e.g.* 2′-fluoro, 2′-*O*-methoxyethyl), as well as non-natural bases. We foresee future studies to understand the nuclease stability of additional chemical modifications. More generally, we believe these data demonstrate that DNA barcodes can be used to study many modifications at once. We anticipate future studies utilizing this platform to study how modifications affect the biodistribution of nucleic acid drugs, including those delivered by biomaterials for targeted genetic therapeutics *in vivo.*

## Materials and methods

### DNA barcoding

91 or 95 nucleotide single stranded DNA sequences were purchased as ultramers from Integrated DNA Technologies (IDT). Barcodes were designed with either zero, three, or five nucleotides on the 5′ and 3′ ends were modified with phosphorothioate linkages and 2′-*O*-methyl modifications to reduce exonuclease degradation and improve DNA barcode stability. To ensure equal amplification of each sequence, the we included universal forward and reverse primer regions on all barcodes. Each barcode was distinguished using a unique 8nt sequence. An 8nt sequence can generate over 4^8^ (65 536) distinct barcodes. We used 8nt sequences designed by to prevent sequence bleaching on the Illumina sequencing machine.

### Mouse serum degradation assay

Normal Mouse Serum (ThermoFisher) was diluted to 10.75% in 1× PBS and 93 μL of 10.75% Mouse Serum was aliquoted into sterile, DNase-free eppendorfs. 7 μL of 100 μM DNA barcode mixture was added to each tube and immediately placed on a heated shaker block at 37 °C for either 1, 3, or 24 hours. At the respective timepoint, the tube was immediately frozen and stored at to –20 °C for future analysis.

### *In vitro* transfection

*In vitro* experiments were performed using HEK293T cells cultured in DMEM/F-12 50/50 media (Corning) supplemented by 10% (v/v) FBS (VWR) and 1% (v/v) penicillin–streptomycin (VWR). Cells were seeded in a 6-well plate at a density of 300k cells per well. 24 hours later, DNA barcode mixture and Lipofectamine 2000 (ThermoFisher) were added with a total DNA dose of 100 ng per well. 24 hours later, DNA was isolated using 50 μL of QuickExtract (EpiCentre).

### *In vivo* nanoparticle formulation

Nanoparticles were formulated using a microfluidic device as previously described.^25^ Briefly, nucleic acids (DNA barcodes) were diluted in 10 mM citrate buffer (Teknova) while lipid–amine compounds and alkyl tailed PEG (Avanti Lipids) were diluted in ethanol. Citrate and ethanol phases were combined in a microfluidic device by syringes (Hamilton Company) at a flow rate of 600 μL min^–1^ and 200 μL min^–1^, respectively. Nanoparticles were dialyzed in 1× PBS for 2 hours before sterile filtration.

### Animal experiments

All animal experiments were approved by (and performed in accordance with) the Georgia Institute of Technology IACUC committee, and all experiments were compliant with the relevant laws and institutional guidelines. C57BL/6J (#000664) mice were purchased from The Jackson Laboratory and used between 5–8 weeks of age. In all *in vitro* and *in vivo* experiments, we used *N* = 3–4 group. Mice were injected intravenously *via* the lateral tail vein. The nanoparticle concentration was determined using NanoDrop (Thermo Scientific). Mice were administered at a DNA barcode dose of 0.5 mg kg^–1^.

### Cell isolation & staining

Cells were isolated 24 hours after injection with LNPs. Mice were perfused with 20 mL of 1× PBS through the right atrium. Tissues were finely cut, and then placed in a digestive enzyme solution with Collagenase Type I (Sigma Aldrich), Collagenase XI (Sigma Aldrich) and hyaluronidase (Sigma Aldrich) at 37 °C at 550 rpm for 45 minutes. The digestive enzyme for heart and spleen included Collagenase IV. Cell suspension was filtered through 70 μm mesh and red blood cells were lysed. Cells were stained to identify specific cell populations and sorted using the BD FacsFusion cell sorter in the Georgia Institute of Technology Cellular Analysis Core. Cell populations were labeled with anti-CD31 (390, BioLegend) and anti-CD45.2 (104, BioLegend), we define lung endothelial cells as CD31^+^CD45^–^.

### PCR amplification for illumina sequencing

All samples were amplified and prepared for sequencing using a two-step, nested PCR protocol. More specifically, 2 μL of primers (10 μM for base reverse/forward) were added to 5 μL of Kapa HiFi 2X master mix, and 3 μL template DNA/water. This first PCR reaction was ran for 20–30 cycles. The second PCR, to add Nextera XT chemistry, indices, and i5/i7 adapter regions was ran for 5–10 cycles and used the product from ‘PCR 1’ as template. Dual-indexed samples were ran on a 2% agarose gel to ensure that PCR reaction occurred before being pooled and purified using BluePippin (Sage Science).

### Deep sequencing

Illumina sequencing was conducted in Georgia Institute of Technology's Molecular Evolution core. Runs were performed on an Illumina NextSeq. Primers were designed based on Nextera XT adapter sequences.

### Barcode sequencing normalization

Counts for each particle, per cell type, were normalized to the barcoded mixture applied to serum, cells, or injected into the mouse.

### Data analysis & statistics

Sequencing results were processed using a custom R script to extract raw barcode counts for each tissue. These raw counts were then normalized with an R script prior for further analysis. Statistical analysis was done using GraphPad Prism 7; more specifically, one-way ANOVAs were used where appropriate. For all conditions, *n* = 3 unless otherwise stated. Data is plotted as mean ± standard error mean unless otherwise stated.

### Data access

The data, analyses, and scripts used to generate all figures in the paper are available upon requests made to dahlmanlab.org.

## Conflicts of interest

J. E. D. and C. D. S. have filed intellectual property related to this publication.

## References

[cit1] Rizk M., Tuzmen S. (2017). Pharmacogenomics Pers. Med..

[cit2] Lorenzer C., Dirin M., Winkler A. M., Baumann V., Winkler J. (2015). J. Controlled Release.

[cit3] Eder P. S., DeVine R. J., Dagle J. M., Walder J. A. (1991). Antisense Res. Dev..

[cit4] Zagorovsky K., Chou L. Y., Chan W. C. (2016). Proc. Natl. Acad. Sci. U. S. A..

[cit5] Yang X. C., Sullivan K. D., Marzluff W. F., Dominski Z. (2009). Mol. Cell. Biol..

[cit6] Morrissey D. V. (2005). Nat. Biotechnol..

[cit7] Khvorova A., Watts J. K. (2017). Nat. Biotechnol..

[cit8] Renaud J. B. (2016). Cell Rep..

[cit9] Hendel A. (2015). Nat. Biotechnol..

[cit10] Finn J. D. (2018). Cell Rep..

[cit11] Yin H. (2017). Nat. Biotechnol..

[cit12] Li B. (2017). Nat. Biomed. Eng..

[cit13] Han D. (2017). Science.

[cit14] Pei W. (2017). Nature.

[cit15] Shalem O. (2014). Science.

[cit16] Konermann S. (2015). Nature.

[cit17] Chow R. D. (2017). Nat. Neurosci..

[cit18] Platt R. J. (2014). Cell.

[cit19] Chen S. (2015). Cell.

[cit20] Deverman B. E. (2016). Nat. Biotechnol..

[cit21] Paunovska K. (2018). Nano Lett..

[cit22] Dahlman J. E. (2017). Proc. Natl. Acad. Sci. U. S. A..

[cit23] Yaari Z. (2016). Nat. Commun..

[cit24] Conesa A. (2016). Genome Biol..

[cit25] Dahlman J. E. (2014). Nat. Nanotechnol..

